# Mr. Annesley's Topographical and Statistical Reports of the Diseases Most Prevalent in India

**Published:** 1826-10-01

**Authors:** 


*Topographical and Statistical Reports of the Diseases most prevalent in the Different Stations and Divisions of the Army under the Madras Presidency.* By JAMES ANNESLEY, Esq. Madras Medical Establishment, &c. 8vo. 1825.

No doubt can exist as to the great utility of reports like these, especially in a widely extended empire like that of the East, where medical officers and troops are often ordered suddenly to stations with which they are totally unacquainted in a medico-topographical point of view. The utility of such reports, however, are not so completely unalloyed as Mr. Annesley seems to think. The features of a country or a particular station will remain apparently the same, while the prevailing diseases will not only vary, but assume diametrically opposite characters! This fact is so notorious in all countries, that to adduce examples would be waste of time. It ought to check overweening confidence in the most authentic records of the past, and keep us constantly on the watch for that which exists at the moment of our observation. Still it is a great advantage to know what has happened to others in any new place to which we may be sent. History is not useless although no period of it can ever run its course over again.

We cannot touch on the tabular returns and reports of this part of Mr. Annesley's work, but merely glance at the topography and prevalent diseases of the places mentioned below, premising that the period embraced is that between the years 1815 and 1821 inclusive.

*Presidency Division.* The climate of the Carnatic is dry and hot—the thermometrical range from 75 to 92; but in May and June often rising to 98 and 105. The most disagreeable months, Mr. Annesley thinks, are February, March, and April, when the southerly winds blow. May, June, and July are most healthy, but the hot land-winds of this period are certainly very unpleasant. The greatest degree of sickness prevails from August to November, viz. about the change of the S.W. to the N.E. monsoon.

The prevailing diseases are fever, dysentery, and hepatitis. Dysentery, Mr. A. thinks, is more dependent, in this presidency, upon circumstances than upon the climate. These circumstances are, irregular living, hard drinking—probably exposure to the sun.

The proportion of liver diseases being 25 per cent. at Madras, while in the centre and southern divisions they were only 13 to 15, our author endeavours to account for the circumstance by the practice that exists of sending chronic cases of hepatitis from outstations to the Presidency General Hospital. We are not aware, however, why liver cases in particular should be sent to Madras, and till this information is afforded, we cannot but continue to think that the climate of this Presidency is peculiarly prolific of hepatic affections—a fact that has often been observed by former writers.

*Symptoms and Treatment of the Diseases.* Mr. Annesley considers the climate and diseases of the Carnatic (including all that space of country situated between the western hills, and the coast extending from Cape Comorin and the Kisthna river) as perfectly similar, or rather identical.

*Fever*. This is the synocha of Cullen—being continued and inflammatory, and evinced by full hard pulse, hot skin, suffused eyes, head-ache, and general pains. It is easily checked, "if immediately attacked, and boldly treated."

"Bleeding, either general or local, according to circumstances, is a safe remedy. It will always be attended with benefit, and should never be lost sight of. The apprehension that blood is not rapidly made in India, is, I fear a fatal error. Such an opinion never can be maintained upon any just principle, though it is a prejudice that exists in a great degree, even amongst those whose field for practice has been considerable. Such fears being sometimes inculcated by men high in office and in power, they cannot fail to paralyse the hands of medical men on their first arrival in India, and they serve to lay the foundation of erroneous notions of practice. The sooner therefore, the prejudice is pointed out the better, as candid and enlightened observation cannot fail of leading to the adoption of juster views.

"Bleeding I consider, in the first instance, as the sheet-anchor in the treatment of this disease, which, when followed up by active purgation, will, probably, in a few days remove it. But purgatives should, nevertheless, be prescribed as long as there is any degree of excitement in the system, and as long as the dejections are viscid, of a dark colour, or otherwise morbid. When the alvine secretions are altered, and become healthy, an alterative course of medicine may then be had recourse to with the best effects." 265.

*Centre Division*. This includes Vellore, Arcot, Wallajahbad, Poonamallee, Nellore, and Ongole. The sickly seasons are the same as at the Presidency—the climate nearly the same, but the heat is more felt on account of the want of the sea-breezes. Fever and dysentery are the prevailing diseases—especially the latter, which is the scourge of our troops. It rises in some places to 30—37 per cent!

*Dysentery*. Our author divides the disease into two varieties—acute and erythematic. "The first is acutely inflammatory, and if not checked by bold and decided practice, will very soon terminate fatally, or lay the foundation for that chronic stage of dysentery which disables so many men, and is the cause of the great number of discharges annually from the service."

"The cause of dysentery amongst the soldiery in India arises unquestionably more from irregularity and diet than climate; though I admit that, in some instances, the latter is intimately connected with functional derangement of the liver. The sick-list of a regiment is always increased after pay-day, and dysentery is the general disease. The symptoms are well marked: severe pain in the bowels; straining; full, strong pulse; foul, loaded tongue; motions very frequent, and small in quantity; sometimes consisting of morbid, offensive matter, but generally, in the first instance, of mucus with blood: and it is not unusual to see very considerable discharges of blood from the bowels. Upon examining the abdomen, a very considerable fulness and tension, with great tenderness, are observed, and particularly at the caput cæcum and sigmoid flexure of the colon; the tongue is sometimes white and dry, and the pulse quick, small, and irritable, with general febrile excitement.

"The causes of dysentery being very nearly the same as those of fever and hepatitis, the treatment must be in many respects, also, the same. Dysentery is a disease that requires great decision in the treatment, because much is to be done in a few hours; and if it be not got under control in that time, the patient is either lost, or the basis of a broken constitution is laid.

"The indications of cure are to diminish general vascular excitement, to remove acrid and accumulated matter from the bowels, to allay the irritation of these viscera, and to restore them to healthy action. As this disease is entirely confined to the large intestine from the cæcum to the rectum, attention should be directed to that particular seat; and therefore emollient injections should be used frequently, to clear away any matter that may lodge in it. Leeches should be applied in the course of the colon, particularly when there is tenderness of the abdomen. Calomel, in ℈j. doses, will always allay irritation of the stomach, and should therefore be given, and be followed up by oily purgatives. In full plethoric subjects, general bleeding will always be attended with benefit; but in those who have been long in India, I have found leeches answer better, because they diminish action without destroying power, and any quantity of blood may be taken by them.

"So long as pain continues leeches may be applied, and, till the dejections become natural and healthy, the calomel in ℈j. doses should be given every night, and oily or saline purgatives every morning. Injections in the course of the day will be administered with benefit, and after the pain is removed, if a general soreness continue, a large blister over the whole belly will always be useful.

"In the treatment of dysentery, as well as of all other acute diseases in India, the first twelve or twenty hours are of the first consequence, in order to make an impression upon the constitution, and to bring the disease under control, before serious structural derangement has supervened: when these objects are accomplished, the future treatment may be regulated according to circumstances.

"Local pains may be removed by the occasional application of leeches. Tenesmus, which depends upon inflammation or irritation of the rectum, will be alleviated by anodyne enemas, not exceeding ℥iss. or ℥ij. which will generally remain in the bowel; but calomel, as a purgative, with oily and sometimes saline laxatives, must be prescribed till the secretions assume a healthy appearance, when tonic laxatives may be given, and continued till the cure is completed." 278.

The erythematic form of dysentery is much more obscure, and consequently more dangerous. The symptoms are, a dull and deep-seated pain in the bowels, sufficient to distress a patient, but not so severe as to excite alarm. There is no external pain—no febrile excitement—alvine discharges morbid and acrid. Mr. Annesley considers the disease as a sub-acute inflammation of the mucous membrane of the colon. "If not treated successfully, it runs into ulceration throughout the whole intestine."

"Full doses of calomel, with such other purgatives as act upon the mucous glands, are required here, and should be continued without intermission, till healthy action is produced. Leeches, and blisters over the abdomen, are always attended with benefit; and the purgative I have found to answer best in combination with calomel, is castor oil and the following bitter aperient mixture,* quickened occasionally by two or three drachms of sulphate of magnesia." 279.

*Northern Division.* This takes in a tract of country extending from the Kisthna river south, to Ganjam north, on the the Coromandel coast, the principal stations being Masulipatam, Samulcottah, Vizagapatam, Berhampore, &c.

The climate of this district is very pleasant from November till March, the thermometer generally ranging from 72 in the morning, to 88 and 90 in the day. The north-east monsoon being superseded by the S. W. Clouds of dust are flying from the end of March till May. In May and June the land-winds commence, and blow with more or less force till the end of the monsoon. The prevailing diseases are here also, fever and dysentery, which prevail most between August and November, the other months being generally healthy. Excepting Masulipatam and Ellore, the other stations in this district are in the neighbourhood of mountains, thick jungle, and sometimes marshes, where fever of a formidable nature occasionally prevails to a great extent.

*Fever.* The type is bilious remittent—sometimes taking the quotidian and double tertian forms, with certain symptoms of typhus, as stupor, black, dry tongue, and great debility—but without any contagious property. It is commonly called the

"* Infus. Gentian. ℥xij.

Infus. Sennæ, ℥vj.

Tinct. Cardam. ℥ij."

hill or jungle fever. When not completely eradicated, it always terminates in intermittents that come on at particular periods of the moon, often continuing for years to harrass its unhappy victims, both in India and Europe.

At Masulipatam and Ellore, situated in an open country and sandy soil, the fever is by no means so formidable, though of a similar kind. It is readily removed by purgatives. The treatment is the same as has been before recommended—being active depletion early in the disease.

We must pass rapidly over the minor divisions, only noticing the prominent features of prevailing disease.

*Travancore Division.* Liver and dysentery affect the Europeans—ulcers and fevers the natives. The dysentery is generally dependent on the hepatic affection.

"The symptoms of liver disease are very insidious, and not easily detected, particularly when the parenchymatous texture of the viscus is the part affected. The strictest attention, therefore, as well as professional tact, is, perhaps, more required in the management of this, than in any other disease in India; and although it is a difficult task to point out clearly what close observation and pathological research can best disclose, yet there are some symptoms that may be depended upon, and those I shall endeavour to state briefly.

"Acute pain is present only when the coats of the liver are the seat of disease. This symptom is generally attended with febrile heat, full, quick pulse, and white, excited tongue; which are often the only signs that indicate inflammation of the internal structure of the organ. To remove these derangements, general and local bleeding, with smart purgatives, should be resorted to without loss of time, and repeated until they are completely subdued; after which an alterative course of mercury, for a fortnight or three weeks, will effect a cure. This acute form of hepatitis usually affects healthy, robust men, on their first arrival in India, and frequently terminates rapidly in abscess, if not checked at its commencement; the practice, therefore, should be energetic, in order to subdue inflammatory action at the onset of the disease.

"The chronic stage of diseased liver generally occurs amongst old, debilitated Europeans, and amongst those who have suffered from the first or inflammatory stage, and who, having been discharged from medical treatment, had returned to their duty before this organ had recovered a healthy action. This practice of premature dismission from treatment is, I am sorry to say, too common throughout India. It is in the highest degree injurious to the constitution, and it cannot fail to load the pension and invalid establishments. This is the stage of liver disease in which mercurial action is required; and this action is always most beneficially produced when mercurial remedies are exhibited as alteratives and laxatives.

"To affect the mouth has frequently been considered the great desideratum; but as there are many subjects in which ptyalism cannot be produced, to follow up a course of medicine for months, in order to attain an object that may be quite impossible, cannot fail of proving highly injurious to the constitution. Hence the danger and mischief of an indiscriminate use of mercury in the diseases of India." 291.

We do not agree with our author that mercury is more beneficial in chronic, than in acute dysentery. On the contrary, we are well convinced that this medicine is, upon the whole, more effectual in acute, than in chronic diseases of every kind. *With it*, the most active depletion should be combined, no doubt—but *without it*, the most active depletion will often fail in the acute dysentery and hepatitis of hot countries. This is our conviction. Let a fair and unprejudiced trial be made of the comparative merits of the two methods—and then let the practitioner judge for himself.—

We cannot, indeed, bring ourselves to imagine that, in these days of anti-mercurial outcry and prejudice, there will be many so lost to all common sense as "to follow up a course of medicine for months in order to obtain an object that may be quite impossible;" but Mr. Annesley has fallen into the now prevailing error of taking the exceptions for the general rules. Because there are individuals who are insusceptible of the salutary operation of mercury in hepatic and dysenteric affections, and others who are severely affected from a small quantity of the medicine, are we to proscribe the medicine, *in toto*, or rail against it in "good set terms," so as to chime in with popular prejudice, and catch the gale of popular favour? Be it so. Truth will, we hope, in the end prevail; and this is the object which we have in view.

Mr. Annesley observes, in the first place, that he is "contending against the bulk of opinion in India;" and in the second place, that he "does not intend to say that mercury is not a most valuable medicine, and indeed one from which more is to be expected than any other, perhaps in the materia medica; but it may be, and it certainly is, upon many occasions, carried far beyond the bounds of judicious administration." We grant this—what then? The fault lies with the *men* and not with the *medicine*. Our author remarks (page 292) that, "when the mouth becomes affected, it is very satisfactory, because it shews that the absorbents and the glandular system are not in a state of torpor; but to continue the use of it for months, or even till ptyalism is produced, I must contend is most injurious to the constitution. I look to its effects on the alvine secretions, and when I see them changed, and find healthy discharges produced, I consider that the use of mercury is beneficial."

Let it be remembered that we are speaking of the acute and chronic hepatitis and dysentery of India—and, bearing this in mind, we appeal to clinical observation whether the alvine secretions are often brought to a healthy condition *before* the gums and breath are affected by mercury. In our own observations, and we are not disposed to give up our opinions when grounded on ocular demonstration, we have generally found the improvement in the secretions and the mercurial odour on the breath either consentaneous, or the latter taking *precedence*. We are perfectly ready then to take Mr. Annesley's criterion for our guide—but let the practitioner take particular notice of the above remark, and if we are wrong let him condemn us.

There is another state of diseased liver, observes Mr. Annesley, which is more common and more obscure than either of those above-mentioned, "and this is a congestive state, in which the *portal* system partakes very largely of the derangement, and which is accompanied by a loaded state of the gall-bladder, and by obstruction in its ducts." The symptoms are, oppression and weight at the præcordia and pit of the stomach, without pain, but with a sense of fulness and distention about the chest, as if there was a heavy weight in the neighbourhood of the stomach. Alterative mercurials, with aloetic purgatives, were the remedies most successful in our author's hands, aided by leeches and warm poulticing to the epigastric region, with frequent frictions of stimulating liniments. "As dysentery may probably depend upon disease of the liver, and as we find in India, that it actually does so depend in many cases, the removal of the *latter* disorder will frequently prevent the *former*."

We do not deem it necessary to accompany our author through his topographical details of some other stations, though they will be found worthy of attention by those whose destinies may lead them into those distant regions. To them we can safely recommend Mr. Annesley's volume as a very valuable addition to their necessarily limited library.

Mr. Annesley promises a work on the Diseases of India generally; and most happy shall we be to give every possible extension of publicity to the labours of so experienced and talented an observer.

